# Circulating Y-RNAs in Extracellular Vesicles and Ribonucleoprotein Complexes; Implications for the Immune System

**DOI:** 10.3389/fimmu.2018.03164

**Published:** 2019-01-15

**Authors:** Tom A. P. Driedonks, Esther N. M. Nolte-'t Hoen

**Affiliations:** Department of Biochemistry and Cell Biology, Faculty of Veterinary Medicine, Utrecht University, Utrecht, Netherlands

**Keywords:** Y-RNA, extracellular vesicles, exosomes, ribonucleoprotein complexes, immune signaling, biomarker

## Abstract

The exchange of extracellular vesicles (EV) between immune cells plays a role in various immune regulatory processes. EV are nano-sized lipid bilayer-enclosed structures that contain a multitude of proteins and small non-coding RNA molecules. Of the various RNA classes present in EV, miRNAs have been most intensively studied because of their known gene-regulatory functions. These miRNAs constitute only a minor part of all EV-enclosed RNA, whereas other 20–200 nt sized non-coding RNAs were shown to be abundantly present in EV. Several of these mid-sized RNAs perform basic functions in cells, but their function in EV remains elusive. One prominent class of mid-sized extracellular RNAs associated with EV are the Y-RNAs. This family of highly conserved non-coding RNAs was initially discovered as RNA component of circulating ribonucleoprotein autoantigens in serum from Systemic Lupus Erythematosus and Sjögren's Syndrome patients. Y-RNA has been implicated in cellular processes such as DNA replication and RNA quality control. In recent years, Y-RNA has been abundantly detected in EV from multiple different cell lines and biofluids, and also in murine and human retroviruses. Accumulating evidence suggests that EV-associated Y-RNA may be involved in a range of immune-related processes, including inflammation, immune suppression, and establishment of the tumor microenvironment. Moreover, changes in plasma levels of extracellular Y-RNA have been associated with various diseases. Recent studies have aimed to address the mechanisms underlying their release and function. We for example showed that the levels of EV-associated Y-RNA released by immune cells can be regulated by Toll-like receptor (TLR) signaling. Combined, these data have triggered increased interest in extracellular Y-RNAs. In this review, we provide an overview of studies reporting the occurrence of extracellular Y-RNAs, as well as signaling properties and immune-related functions attributed to these RNAs. We list RNA-binding proteins currently known to interact with Y-RNAs and evaluate their occurrence in EV. In parallel, we discuss technical challenges in assessing whether extracellular Y-RNAs are contained in ribonucleoprotein complexes or EV. By integrating the current knowledge on extracellular Y-RNA we further reflect on the biomarker potential of Y-RNA and their role in immune cell communication and immunopathology.

## Introduction

Extracellular vesicles (EV) are 50–300 nm sized lipid bilayer-enclosed vesicles containing proteins and nucleic acids (1), which are released by virtually all cells. All living cells, including archaea, bacteria, and eukaryotes release EV, which suggests that the release of EV is a conserved mechanism of cellular communication (2, 3). EV have been found in many body fluids and have been implicated in several diseases, including immune-related disorders, cancer, neurological disorders and cardiovascular diseases (4–7). Characterizing the protein, lipid, and RNA content of EV is an active area of research. One of the major topics in the field is to delineate how differences in EV composition relate to differences in their function, and to determine whether differences in the protein/RNA content of EV can be used as biomarkers for disease.

**Figure 1:**
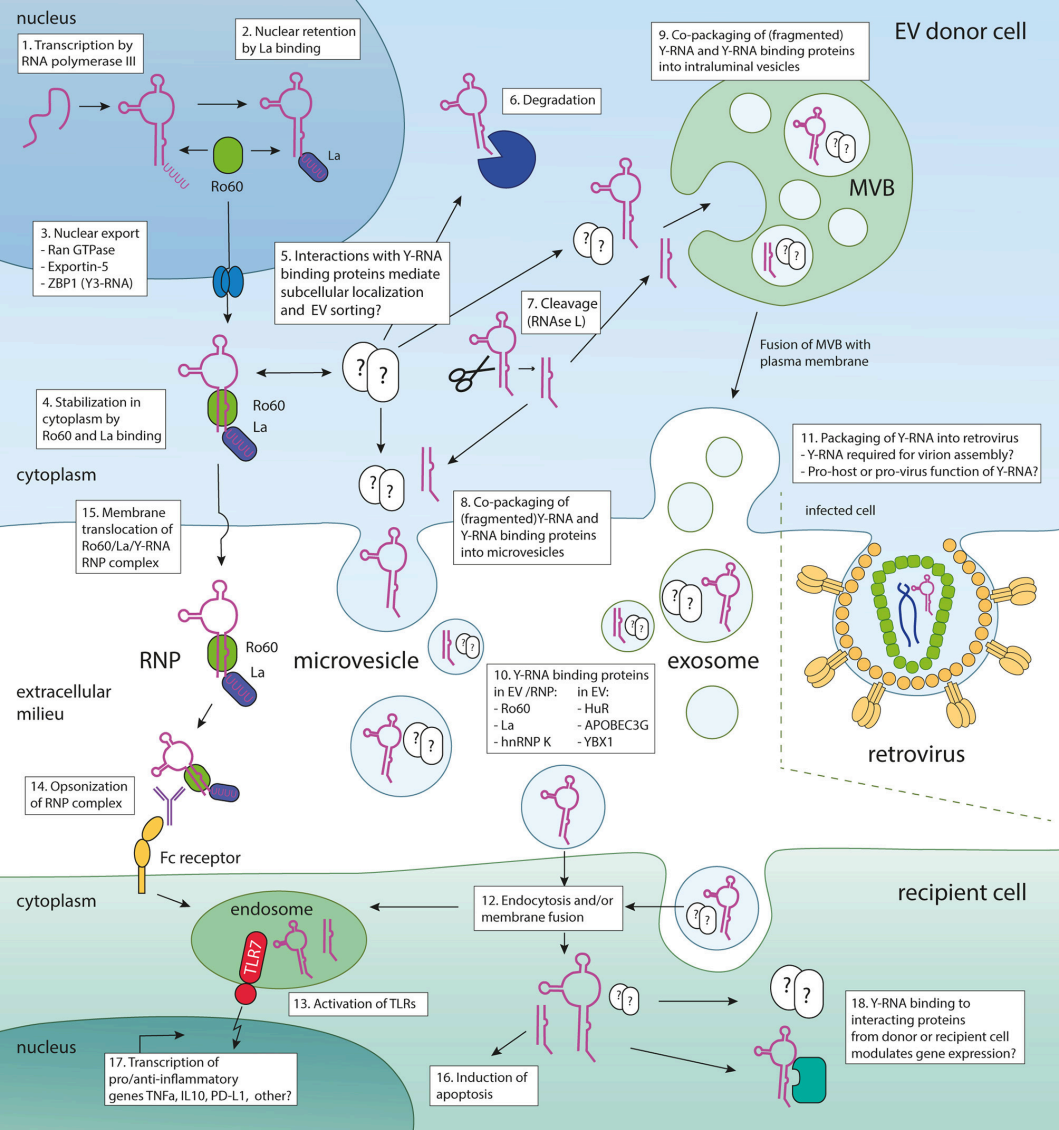
Model illustrating the Y-RNA life cycle, putative pathways for Y-RNA release into the extracellular space, and ideas on the function of Y-RNA transferred to target cells. Various steps in the process of Y-RNA transport within cells and between cells are indicated numerically. Upon transcription (1), newly generated Y-RNA may remain in the nucleus through binding of La (2). Alternatively, upon binding of Ro60, it can be transported into the cytoplasm by Ran GTPase, Exportin-5 and/or ZBP1 (3). In the cytoplasm, Ro60 binding stabilizes Y-RNA (4). Y-RNA can also bind to various other RNA-binding proteins (for instance the Y-RNA binding proteins summarized in Table 3) that may influence its subcellular localization and/or fate (5). Y-RNA may be degraded (6), or be cleaved into fragments by RNAse L (7). Both full-length and fragmented Y-RNA are packaged into EV, either via passive engulfment of Y-RNA by budding membranes, or through protein-mediated shuttling toward sites of EV biogenesis [such as the plasma membrane (8) or late endosomes/multivesicular bodies (9)]. Certain proteins known to bind Y-RNA are co-packaged into EV, but others may only serve to shuttle Y-RNA to the sites of EV biogenesis. The Y-RNA binding proteins from Table 3 that have been found in extracellular space, associated with EV and/or RNP are listed in (10). In the case of retrovirus infected cells, Y-RNA may be additionally released from cells by incorporation into virions (11). EV can be taken up by recipient cells by endocytosis and/or membrane fusion (12). Y-RNA may be delivered to the endosome, where it may activate TLRs (13). TLR triggering also occurs after uptake of opsonized Y-RNA/Ro60/La RNP complexes (14) which may be released from cells after translocation across the cellular membrane (15). Naked Y-RNA has been shown to induce apoptosis (16). TLR triggering of Y-RNA drives the transcription of various pro- and anti-inflammatory cytokines (17). On a more speculative note, transferred Y-RNA could affect the function of recipient cells through the action of Y-RNA binding proteins present in recipient cells or co-transferred by the EV (18). For example, binding to translation enhancer proteins, such as HuR and HuD, may alter mRNA stability and translation efficiency.

It has been shown that EV-enclosed RNAs can be functionally transferred to target cells (8–11). Many studies have focused on elucidating the miRNA composition of EVs because of their known effects on gene regulation. However, miRNAs only constitute a minor percentage of EV-enclosed RNA. In contrast, the majority of EV-RNA consists of other types of small- to mid-sized non-coding RNAs [20–200 nt] (12–16). Of these RNAs, Y-RNA attracted attention because this conserved RNA has been detected in EV from many different cell types and in various vertebrate species (12–14, 16–18). Moreover, Y-RNAs are highly abundant in body fluids, such as blood and seminal fluid (19, 20). Recent data indicate that as much as 67% of sequencing reads in plasma samples of healthy donors map to Y-RNA (19). There are also indications that the levels of Y-RNA in body fluids could correlate with disease (21, 22). Research on the regulation of Y-RNA sorting into EV and the function of EV-associated Y-RNA is in its early days. Our laboratory recently showed that incorporation of Y-RNA in EV released by dendritic cells is regulated by immunogenic and tolerogenic stimuli imposed on these cells (16). Initial studies on the function of EV-enclosed Y-RNA reported pro- and anti-inflammatory effects (23–25). Given the increasing interest in and number of publications on extracellular Y-RNA we took the initiative to compile an inventory of data and assess the inter-study comparability of discoveries in this field. In this review, we provide an overview of reports describing the occurrence of extracellular Y-RNA in EVs from various cell types and biofluids, as well as its signaling properties and potential immune-related functions. After introducing general aspects of EV-associated RNA and the role of Y-RNA inside cells, we summarize current knowledge on Y-RNA association with EV and with extracellular ribonucleoprotein complexes. In addition, we provide an overview of protein partners of Y-RNA that have also been detected in EV and may therefore be involved in sorting these RNAs into EV. Finally, we provide an overview of the proposed functions of extracellular Y-RNA and reflect on its biomarker potential. Key steps in the Y-RNA life cycle, putative pathways for Y-RNA release into the extracellular space, and ideas on the function of Y-RNA transferred to target cells are illustrated in Figure 1.

## Intercellular Communication via EV-associated RNA

EV constitute a unique way in which molecular messages are exchanged between cells. Upon transfer, the lipids, proteins, and RNA associated to EV can modify the function of recipient cells (1, 3, 26–28). EV are either formed by inward budding into multivesicular bodies, which upon fusion with the plasma membrane are released as exosomes, or by direct budding off the plasma membrane (microvesicles) (1, 3). Exosomes and microvesicles cannot be separated using currently available methods, and are therefore collectively referred to as EV. EV are heterogeneous in size and molecular composition, but unique molecular markers to distinguish biologically distinct EV subtypes are yet to be discovered. Various classes of RNA have been detected in EV, including mRNA, lncRNA, circRNA, and small non-coding RNA. Most of the EV-RNA consists of small non-coding RNA types, such as miRNA, tRNA, rRNA, snoRNA, Y-RNA, SRP-RNA (7SL), and Vault RNA (12, 13).

It is important to realize that not all extracellular RNA is associated with EV. Other macromolecular structures in the extracellular milieu, such as ribonucleoproteins (RNPs) and lipoprotein particles, also contain RNA (16, 29–31). These other structures overlap in size and/or density with EV and are therefore frequent contaminants in EV preparations (32, 33). The degree to which some of these contaminants co-isolate with EV depends on the fluid used as source of EV and the applied EV isolation method. The most widely used techniques are ultracentrifugation, size exclusion chromatography (SEC), and density gradient centrifugation, in which particles are separated based on mass, size, or buoyant density, respectively. Ultrafiltration-based methods concentrate particles by molecular weight. Precipitation-based methods, on the contrary, concentrate all macromolecules in solution. The advantages and disadvantages of available methods have been extensively reviewed (32, 34). Sequential application of methods that separate particles based on size and on density likely yields the purest EV preparations.

It has been demonstrated that both the protein- and miRNA composition of EV can change upon exogenous stimuli imposed on EV-producing cells (9, 13, 16). These changes in the “molecular message” that is conveyed via EV can lead to alterations in the function of EV-recipient cells. We have previously shown that, in addition to miRNA, the levels of Y-RNA and snoRNA in EV are regulated by exogenous stimuli imposed on the EV-producing immune cells (16). Importantly, the activation-induced changes in EV-RNA composition we observed only partly reflected changes in cellular RNA, which suggests that the cell stimuli triggered mechanisms for sorting of specific RNA types into EV (16).

Several *in vitro* and *in vivo* studies have demonstrated that intercellular transfer of EV-associated miRNA and mRNA leads to changes in recipient cell function (8, 10, 32, 35–37). For example, EV-mediated transfer of miR-155 and miR-146a from wildtype dendritic cells to recipient cells deficient for these miRNAs modulated the response of these recipient cells to lipopolysaccharide (LPS). Transfer of miR-155 into miR-155 negative recipient cells increased IL6 release via repression of SHIP1 and BACH1, while transfer of miR-146a dampened this LPS response by repression of TRAF6 and IRAK1 (10). Functional transfer of mRNA was evidenced by demonstrating that EV-associated mRNA derived from *in vitro* cultured mast cells could be translated in recipient cells (8). *In vivo* evidence for EV-mediated transfer of mRNA was provided by the use of Cre-Lox mouse models. Hematopoietic cells or tumor cells expressing Cre-recombinase were shown to release EV containing Cre-mRNA, which induced recombination-mediated expression of floxed fluorescent reporter genes in recipient cells at local or distant sites (36, 38). The functional effects of other RNA classes, which compose the major part of all EV-RNA, are beginning to be unveiled. The experimental approaches used to study miRNA transfer may serve as a basis to gain understanding of how other EV-associated RNA classes affect recipient cell behavior, but these RNAs likely exert their functions via mechanisms other than base-pairing with RNA targets. Although many questions remain to be answered, EV-mediated transfer of RNA appears to be a common, frequent, and adaptable process that cells employ to communicate with other cells.

## Intracellular Location and Function of Y-RNAs

In order to unravel the role of Y-RNA in EV, it is important to understand the function of Y-RNA inside cells. Y-RNAs have been studied for many years and multiple comprehensive reviews are available on this topic (39–44). Y-RNAs were initially discovered as RNA components of circulating ribonucleoprotein (RNP) autoantigens Ro60 and La in serum from lupus patients (45). These RNP are major targets for autoimmune responses in rheumatic diseases such as Systemic Lupus Erythematosus (SLE) and Sjögren's Syndome (SS) (46, 47). Y-RNAs are well-conserved through evolution and have been found in all vertebrate species (48, 49), and related ncRNAs have been found in some bacteria (44) and in nematodes (50, 51). Although the nematode ncRNAs called “stem-bulge RNAs” resemble Y-RNA because of their stem-loop structure (51), they differ from bona fide Y RNAs in that they have not been found complexed with Ro60 in cells (50). The human genome encodes four different Y-RNAs (hY1, hY3, hY4, and hY5) while only two different Y-RNAs exist in rodents (mY1 and mY3) (52). All Y-RNAs contain a long stem, formed by basepairing the 5′ and 3′ ends, that contains the Ro60 binding site, but individual Y-RNAs differ slightly in their primary and secondary structures (53).

Y-RNAs are transcribed in the nucleus by RNA polymerase III (54) (Figure 1, box 1). Binding of La to the 3′ oligo-uridine tail of Y-RNA mediates its nuclear retention and protects Y-RNA from 3′ to 5′ exonucleolytic degradation (55, 56) (Figure 1, box 2). Binding of Ro60 to the stem region of Y-RNA enhances nuclear export (55), which is mediated by Ran GTPase and exportin-5 (57) (Figure 1, box 3). Y3-RNA can also be exported via an alternative pathway through binding of Ro60/Y3-RNA to zipcode binding protein (ZBP1), enabling export via exportin-1 (alternatively named CRM1) (58). It is not fully understood whether Y-RNA is transported from the nucleus in complex to La, or whether La reassociates to Y-RNA after nuclear export. Binding of Ro60 stabilizes Y-RNA in the cytoplasm (Figure 1, box 4), as knockout of Ro60 was shown to drastically reduce Y-RNA levels (59). The loop of Y-RNA is known to interact with various other proteins including nucleolin (60), polypyrimidine tract-binding protein (PTB/hnRNP I) (61), and zipcode-binding protein 1 (ZBP1) (62). It has been proposed that interactions with these proteins could affect the localization and/or function of Y-RNA (43) (Figure 1, box 5). Conversely, Y-RNA can influence the localization of Y-RNA binding proteins, since siRNA-mediated knockdown of Y-RNA leads to nuclear accumulation of Ro60 (63).

Various housekeeping functions of Y-RNA have been described, such as involvement in DNA replication (43) and quality control of non-coding RNA (64, 65). The effects of Y-RNA on DNA replication were first observed in cell-free reactions, in which addition of purified Y-RNA subtypes increased the percentage of dividing nuclei (66). siRNA-mediated knockdown of Y1-RNA in cells was sufficient to reduce the percentage of cells in S-phase, during which DNA replication takes place (66). In a later study, association of Y-RNA with chromatin was shown to increase 2–4 fold during S-phase and to decrease during G1 phase and mitosis, which suggests an association with the origin replication complex (ORC) (67). It has been shown that a specific sequence in the Y-RNA stem was sufficient to increase DNA replication in cell-free reactions (68). However, Ro60 knockout cells that contain ~30-fold lower Y-RNA levels did not show reduced growth rates (59). The exact molecular mechanisms by which Y-RNA affects DNA replication therefore remain unresolved.

Y-RNAs are involved in regulating the degradation of misfolded RNAs through its interaction with Ro60 (47, 64, 69). Misfolded RNAs that contain a 3′ single-stranded end and adjacent helices can bind Ro60 (70, 71). This has been shown for 5S rRNA in *Xenopus* (72) and for U2 snRNA in mouse embryonic stem cells (59), and suggested for a wider variety of structured RNAs (70). Structural analyses revealed that the single-stranded tail of the misfolded RNAs extend through the Ro60 cavity, while helices bind on its outer surface (70). Y-RNAs sterically blocked binding of misfolded RNAs to Ro60, thereby regulating the RNA quality control function of Ro60 (64). A similar mechanism has also been demonstrated for the bacterial ortholog of Ro (*r*o-*s*ixty *r*elated, Rsr) (71). In the bacterium *Deinococcus radiodurans* Y-RNA tethers Rsr to the exonuclease PNPase, thereby forming a RNA-degrading RNP complex resembling the eukaryotic exosome (71). It was proposed that tethering to Rsr potentiates PNPase to specifically degrade structured RNAs. Although mammalian PNPases localize inside mitochondria, it has been proposed that Y-RNAs could potentially tether Ro60 to other proteins involved in RNA metabolism, including exoribonucleases, helicases or RNA chaperones (40).

Interestingly, it was recently discovered in neuronal cells that Y3-RNA can act as a molecular sponge for the enhancer protein HuD (ELAVL4) (73). HuD can enhance gene expression by binding and stabilizing the 3′ untranslated regions (UTRs) of specific mRNAs involved in motor neuron differentiation and axonogenesis. This activity is counteracted by Y3-RNA binding to HuD, which leads to changes in HuD localization and reduced expression of the involved mRNAs (73). Moreover, dysregulation of Y-RNA binding to HuD has been found to cause alternative splicing in neurons of Alzheimer patients (74).

In cells, Y-RNA does not only occur in its full length form, but has also been shown to be cleaved into specific fragments of 25–35 nt. This cleavage, which is carried out by the enzyme RNase L (75) (Figure 1, box 7) occurs in response to UV irradiation or by polyI:C-mediated activation of the innate immune system (76, 77). Because Y-RNA fragments arise from conserved ends of the Y-RNA hairpin and have comparable sizes to miRNAs, it was proposed that Y-RNA fragments function similar to miRNAs (78). Although interactions of Y-RNA fragments with Argonaute have been demonstrated, mRNA reporter constructs could not be repressed by Y-RNA/Argonaute complexes (79).

Taken together, the highly conserved family of Y-RNAs interacts with, and regulates the localization and activity of various RNA-binding proteins involved in basic cell functions.

## Y-RNAs Are Abundantly Present in the Extracellular Milieu

RNA sequencing studies aiming to characterize the small transcriptome of EV have indicated that cells release Y-RNAs into the extracellular milieu (12–18, 20, 24, 25, 31, 80). There is now strong evidence that Y-RNAs are abundantly present both in supernatants of multiple *in vitro* cultured primary and immortalized cell types, as well as in various biofluids (see Table 1). In fact, Y-RNA was found to be the most abundant non-coding RNA species in plasma from healthy individuals (19). Multiple studies reported a strong enrichment of Y-RNA in EV relative to intracellular levels, which suggests that the shuttling of Y-RNA into EV is highly efficient (12–14, 25, 31).

**Table 1:** Overview of RNA sequencing studies reporting the presence of extracellular Y-RNA in *in vitro* cell cultures or in body fluids.

**References**	**Sample type**	**EV-enrichment**	**RNA size selection?**	**Y1**	**Y3**	**Y4**	**Y5**
Cambier et al. (24)	Cardiosphere derived cells (CDC)	EV precipitation	No	2	3	1	4
Haderk et al. (25)	Chronic leukemic lymphocytes	UC pellet (100,000 g)	No	2	4	1	3
Kaudewitz et al. (81)	Platelet rich and platelet poor plasma	No	No	3	4	1	2
Dhahbi et al. (82)	Plasma	No	No	n.d.	n.d.	1	n.d.
Vojtech et al. (20)	Seminal fluid	UC pellet (100,000 g)	No	4	3	1	2
Tosar et al. (14)	MCF7 and MCF-10A breast cancer cell lines	UC pellet (100,000 g)	<60 nt	3	2	1	4
van Balkom et al. (17)	Human endothelial cells	Density gradient	No	2	4	2	1
Chakrabortty et al. (80)	K562 myelogenous leukemia and BJ primary fibroblast	EV precipitation	<200 nt	-	-	-	1
Repetto et al. (22)	Primary macrophages	No	No	3	4	2	1
Shurtleff et al. (15)	HEK293T cell line	Density gradient	No	3	1	2	4
Wei et al. (31)	Glioblastoma cell line	Ultrafiltration	<65 nt	1	4	2	3
Driedonks et al. (16)	Primary bone-marrow derived dendritic cells (mouse)	Density gradient	<275 nt	2	1	Not in mouse	Not in mouse
Lunavat et al. (18)	Melanoma cell lines	UC pellet (100,000 g)	<175	Not specified			
Nolte-'t Hoen et al. (12)	DC - T cell co-cultures (mouse)	UC pellet (100,000 g)	<70 nt	Not specified			
Bellingham et al. (13)	Neuronal cells (mouse)	UC pellet (100,000 g)	<150 (incl adapters)	Not specified			
Yeri et al. (19)	Plasma, saliva, urine	No	n.s.	Not specified			

*Different Y-RNA subtypes are ranked based on their relative abundance reported by each study. The Y-RNA subtype with the highest RPM value in a study is ranked with 1, the second highest RPM value as 2, etc. The column ‘EV-enrichment’ indicates the method that was used to concentrate EVs from supernatant/biofluid. n.d., not determined, UC, ultracentrifugation*.

The frequent detection of Y-RNAs in the external milieu of cells suggests that release of Y-RNAs from cells is a common and ubiquitous process. We compared the abundance of Y-RNA subtypes reported in each of the RNA sequencing studies and ranked these from 1 (highest) to 4 in Table 1. Although differences exist between studies that used different cell types or EV-purification methods, Y4 is most abundantly detected in the extracellular milieu. A number of studies indicate that differences exist between the relative abundance of Y-RNA subtypes inside cells and those released by these cells into the extracellular milieu (14, 25, 31), which supports subtype-specific differences in Y-RNA release.

The data in Tables 1, 2 indicate that different size selections were applied during sequencing library generation for extracellular Y-RNA detection. Several of the studies primarily focused on miRNA detection and therefore applied a narrow size selection (<65 nt). This hampers detection of longer transcripts such as full-length Y-RNA, which are 83–110 nt in size. The sequencing approach in these studies may therefore bias toward detection of Y-RNA fragments (12, 14, 19, 31). However, Y-RNA fragments have also been detected in sequencing studies where no size selection was applied (Table 2) (17, 19, 20, 25, 80–82). Most studies show that the extracellular Y-RNA fragments derive from both the 5′ and 3′ arms of the Y-RNA hairpin and that they can be categorized in defined lengths of ~21 nt, ~30 nt and ~40 nt (Table 2). Fragments of the 5′ arm of the Y-RNA hairpin were generally found to be more abundant than the 3′ fragments. Although these data suggest that Y-RNA fragments are frequently released from cells, reliable detection of full-length Y-RNA in these studies may have been hampered by technical limitations. Y-RNA forms complex RNA structures that are known to negatively influence cDNA synthesis efficacy and to introduce bias in deep sequencing. Reverse transcriptases may not efficiently read through these complex RNA structures, leading to overestimation of fragmented non-coding RNA in sequencing data (83, 84). This is corroborated by recent sequencing studies deploying reverse transcriptases that are insensitive to secondary structures, which detected mostly full-length Y-RNAs (and other structured ncRNA such as tRNA) (15, 85). By using Northern blot analysis, we also recently confirmed that EV contain mostly full-length Y-RNA and only a small amount of 19–35 nt fragments (16). This urges caution in drawing conclusions on the presence of Y-RNA fragments in EV based on RNA sequencing data (16). Taken together, both full-length and fragmented forms of extracellular Y-RNA are abundantly detected in body fluids and in culture supernatant of various cell lines.

**Table 2:** Overview of studies reporting the presence of Y-RNA fragments by RNA sequencing analysis of extracellular RNA.

**References**	**sample type**	**Sequencing method**	**Size selection (nt)**	**Y-RNA fragments in sequencing**	**5^**′**^ length (nt)**	**3^**′**^ length (nt)**	**Fragment detected by Northern blot**
van Balkom et al. (17)	HMEC	Illumina smallRNA	No	Y1, Y4, Y5	30–39	19 and 33	No
Cambier et al. (24)	Cardiosphere derived cells (CDC)	Ion Total RNA seq	No	Y1, Y3, Y4, Y5	n.s.	n.s.	No
Chakrabortty et al. (80)	K562 myelogenous leukemia and BJ primary fibroblast	Illumina TruSeq SmallRNA	20–200	Y5	23, 29, 31	31	Y5 5p
Dhahbi et al. (82)	Plasma	Illumina TruSeq smallRNA	No	Y4	27, 30–33	-	Y4 5p
Dhahbi et al. (21)	Plasma (healthy vs. cancer)	Illumina TruSeq smallRNA	No	Y4	30-33	25 - 29	Y4 5p
Driedonks et al. (16)	Primary bone-marrow derived dendritic cells (mouse)	NebNext smallRNA	15–275	Y1, Y3	30	21	Y1 5p and 3p
Haderk et al. (25)	Chronic leukemic lymphocytes	NebNext smallRNA	No	Y4	30-32	-	Y4 5p
Kaudewitz et al. (81)	Platelet rich and platelet poor plasma	Illumina smallRNA	No	Y1, Y3, Y4, Y5	-	-	No
Nolte-'t Hoen et al. (12)	DC - T cell co-cultures (mouse)	SOLiD Small RNA Expression Kit	20–70	Yes, but not specified which subtypes	-	-	No
Repetto et al. (22)	Primary macrophages	NebNext Small RNA	25–40	Y4	-	-	Y1 5p
Tosar et al. (14)	MCF7 and MCF-10A cell lines	NebNext smallRNA	<60 nt	Y4	30–33	30−33	No
Vojtech et al. (20)	Human seminal fluid	ScriptMiner smallRNA seq	No	Y1, Y3, Y4, Y5	30–33	-	No
Wei et al. (31)	Glioblastoma	NEBnext smallRNA	15–65 nt	Y1, Y4, Y5	32 nt	-	No
Yeri et al. (19)	Plasma, saliva, urine	Illumina TruSeq	Not specified	Yes, but not specified which subtypes	Not specified	-	No

*Indicated are the Y-RNA subtypes from which the fragments derived, the reported fragment length, and whether the presence of Y-RNA fragments was confirmed by Northern blot analysis*.

## Y-RNA Binding Proteins Identified in EV

Several different proteins are known to interact with Y-RNA inside cells and determine its function or localization (see chapter 2). Additionally, protein binding may shield motifs in Y-RNA which may trigger cellular RNA sensors. For instance, the La-protein potentially shields the triphosphate moiety (56), which may prevent activation of RIG-I (86). Ro60 covers the stem-motif (87), which may prevent activation of dsRNA sensor TLR-3. In the context of EV release, protein partners of Y-RNA may be involved in shuttling of the RNA into EV and in functional effects of transferred Y-RNA in target cells. It is largely unknown which protein partners are associated with Y-RNA in EV and whether this differs between EV of different cellular origin (Figure 1, box 8 and 9). We therefore composed a list of known Y-RNA protein partners and evaluated whether these proteins have been detected in EV by searching public databases of mass spectrometry data of EV-associated proteins (Vesiclepedia) (88). The list of known Y-RNA protein partners can be found in Table 3. Most of these proteins have been identified by immunoprecipitation with antibodies against RNA binding proteins followed by Y-RNA detection, or by using tagged Y-RNA molecules to pull down proteins from cell lysates that directly interact with this RNA (RNA affinity purification). Studies that initially discovered the interaction between an RNA-binding protein and Y-RNA subtypes, as well as later studies further validating this interaction have been listed in Table 3. Twenty-three proteins have been reported to directly interact with Y-RNA. Ro60 and La, which are the best characterized protein partners of Y-RNA, have been discussed in chapter 2. Many of the other Y-RNA-binding proteins (hnRNP I, hnRNP K, RoBPI, ZBP1, YBX1, YBX3, ELAVL1 (HuR), CPSF1, CPSF2, FIPL1 SYMPK, and HuD) function in processing or splicing of mRNA transcripts. Several of these proteins mediate 3′ end processing of human histone-H3 mRNA in conjunction with a truncated form of Y3-RNA called Y3^**^ (89). As mentioned earlier, the protein HuD is specifically expressed in neuronal cells where it enhances translation efficiency by stabilizing the mRNAs of mTORC1-responsive genes, which is counteracted by Y3-RNA binding to HuD (73). Similarly, the related protein HuR, also known to bind Y3-RNA, can bind AU-rich elements in mRNA transcripts. Via this mechanism, HuR was for example shown to influence cytokine production, evidenced by increased interferon-β expression in synoviocytes of arthritis patients, and reduced production of inflammatory cytokines including TNFα and TGFβ in LPS-treated macrophages (95, 96). Two other proteins, MOV10 and Argonaute, are important players in miRNA-mediated gene silencing. Additionally, a number of Y-RNA interacting proteins are involved in virus infection or innate immunity, such as MOV10, APOBEC, IFIT5, SYMPK, YBX1. Interestingly, not all proteins were found to interact with all four human Y-RNA subtypes. This suggests specialized functions for different Y-RNA subtypes, dependent on their associated proteins.

**Table 3:** Overview of proteins known to interact with Y-RNA, as identified by immunoprecipitation or RNA affinity purifications.

**References**	**Protein name**	**Protein function in cells**	**Method to identify Y-RNA binding proteins**	**Y1**	**Y3**	**Y4**	**Y5**	**Entry in vesicle pedia**	**Source of EV**
Hendrick et al. (45); Köhn et al.(89)	Ro60	Binds misfolded RNA	Immunoprecipitation (anti-Ro60), RNA affinity purification	++	++	++	++	Yes	Blood/cell lines
Hendrick et al. (45); Köhn et al.(89)	La (SSB)	Binds 3′ poly-(U) tail of RNA pol III transcripts	Immunoprecipitation (anti-La), RNA affinity purification	++	++	++	++	Yes	Cell lines
Fouraux et al. (60)	Nucleolin	Associates with intranucleolar chromatin	Immunoprecipitation (anti-Ro60 and anti-La)	++	++	−	−	Yes	Blood/urine/cell lines
Fabini et al. (61)	hnRNP I (PTBP1)	Pre-mRNA splicing	RNA affinity purification	++	++	−	−	No	–
Fabini et al. (61); Köhn et al.(89)	hnRNP K	Pre-mRNA binding	RNA affinity purification	++	++	−	−	Yes	urine/cell lines
Thomson et al. (79)	Ago	RNAi mediated gene-silencing	Immunoprecipitation (anti-Ago)	n.d.				No	–
Cheng et al. (90)	Calreticulin	Calcium-binding chaperone	Electrophoretic mobility shift assay (EMSA)	++	++	++	++	Yes	Blood/pleural effusions/saliva/ urine/cell lines
Hogg and Collins (69)	L5 (RPL5)	Component of ribosome	RNA affinity purification	−	−	−	++	Yes	Saliva/urine/cell lines
Bouffard et al. (91); Hogg and Collins (69)	RoBPI (PUF60)	Pre-mRNA splicing, apoptosis and transcription regulation	Yeast three-hybrid assay, immunoprecipitation, RNA-immunoprecipitation (Ro60)	++	++	−	++	Yes	Cell lines
Hogg and Collins (69)	IFIT5	Interferon-induced RNA binding protein, senses viral 5′triphosphorylated RNA	RNA affinity purification	n.d	n.d.	n.d.	++	Yes	Urine/cell lines
Köhn et al. (58); Sim et al.(62); Kohn et al. (89)	ZBP1 (IFGB2P1, IMP1)	Recruits mRNAs to protein-RNA complexes, allowing mRNA transport and transient storage	Immunoprecipitation (anti-Ro60-FLAG), RNA affinity purification	+	++	n.d.	n.d	Yes	Cell lines
Sim et al. (58); Köhn et al. (89)	YBX1	Regulation of mRNA transcription, splicing, translation and stability	Immunoprecipitation (anti-Ro60-FLAG), RNA affinity purification	++	++	++	+	Yes	Cell lines
Sim et al. (58)	YBX3	Binds to GM-CSF promoter. Also binds full-length mRNA and short RNA	Immunoprecipitation (anti-Ro60-FLAG)	n.d				No	–
Sim et al. (58)	MOV10	Required for miRNA-mediated gene silencing. Involved in human hepatitis delta virus transcription	Immunoprecipitation (anti-Ro60-FLAG)	n.d.				Yes	Urine/cell lines
Yamazaki et al. (92); Köhn et al. (89)	Matrin-3	Nuclear matrix protein, nuclear retention of RNA, involved in antiviral response	RNA affinity purification	++	++	−	−	Yes	Urine/cell lines
Köhn et al. (89)	ELAVL1 (HuR)	Stabilizes mRNA and regulates translation	RNA affinity purification	−	++	−	−	Yes	Cell lines
Köhn et al. (89)	CPSF1	Involved in mRNA poly-adenylation	RNA affinity purification	++	++	−	−	Yes	Cell lines
Köhn et al. (89)	CPSF2	Involved in mRNA poly-adenylation	RNA-immunoprecipitation	++	++	−	−	Yes	Cell lines
Köhn et al. (89)	FIP1L1	Involved in mRNA poly-adenylation	RNA-immunoprecipitation	++	++	−	−	No	–
Köhn et al. (89)	SYMPK	Histone mRNA 3′-end processing	RNA-immunoprecipitation	++	++	+	+	Yes	Urine/cell lines
Bogerd et al. (93); Apolonia et al. (94)	APOBEC3G	Inhibitor of retrovirus replication, broad antiviral activity	RNA-immunoprecipitation	n.d.				Yes	Cell lines
Bogerd et al. (93); Apolonia et al. (94)	APOBEC3F	Inhibitor of retrovirus replication, broad antiviral activity	RNA-immunoprecipitation	n.d.				Yes	Cell lines
Tebaldi et al. (73)	HuD (ELAVL4)	Translational enhancer of mTORC1-responsive genes, regulation of mRNA abundance and alternative splicing	RNA-immunoprecipitation	−	++	−	n.d.	No	–

*Indicated are known functions of the human variants of these proteins. Binding to the different Y-RNA subtypes is indicated with +, − and ++, or not-determined (n.d.). Furthermore, identification of these proteins in EV is indicated based on entry in the Vesiclepedia database of EV-associated proteins, as well as the source of EV in which these proteins were detected*.

Next, we searched Vesiclepedia (www.microvesicles.org), a repository for extracellular vesicle proteomics data (88), to investigate which of the known Y-RNA binding proteins have been detected in EV. Interestingly, 18 out of 23 known Y-RNA binding proteins were reportedly present in EV from various cell types (Table 3). In addition, 10 of these proteins have been detected in EV from biofluids such as blood and urine. Of note, most entries in Vesiclepedia are based on mass-spectrometry, which may be prone to false-positive identification of proteins due to its high sensitivity. Therefore, we additionally searched the literature to determine whether the presence of these proteins in EV has been validated by Western blot detection. This was the case for 6 proteins: Ro60 (20, 31), La (31), hnRNP K (97), YBX1 (98), APOBEC3G (99), and ELAV1 (HuR) (100, 101). In studies reporting the presence of Ro60 (20, 31), La (31), and hnRNP K (97), EV had only been enriched by ultracentrifugation/ultrafiltration. These proteins may therefore be associated to EV or RNP or both. Association of HuR and APOBEC3G to EV was convincingly demonstrated using EV purification by density gradient centrifugation (99, 101). Additionally, it was shown that lipid membrane-enclosed YBX1 was protected from protease degradation, indicating that this protein is found inside EV (98).

We noticed that several of the Y-RNA-binding proteins detected in EV have previously been implicated in sorting of miRNAs into EV. ELAV1 (HuR), for example, dissociates miRNA-122 from AGO2/mRNA complexes in hepatocytes and drives subsequent expulsion of miR-122 from the cell via EV (101). Furthermore, YBX1 was shown to package miR-223 into EV from HEK293T cells (102). Initial evidence suggests that YBX1 also plays a role in sorting Y-RNA into EV (15). Knockout of this protein in HEK293T cells resulted in a reduced packaging of Y-RNA into EV. However, disruption of YBX1 did not completely abolish Y-RNA packaging, suggesting the involvement of additional proteins in this process. Moreover, YBX1 knockout also affected the packaging of other small non-coding RNAs such as tRNAs and Vault RNA, which suggests a more general function in EV-RNA packaging. Delineating the mechanisms underlying sorting of RNAs into EV is an area of intense research. Identification of proteins that specifically interact with EV-RNAs of interest, as performed above for Y-RNA, is a starting point to investigate potential involvement in sorting these RNAs into EV. Besides the assumed involvement of RBP in this process, sorting of RNAs into EV may also be influenced by the presence of specific motifs, modifications, or structures in RNA, post-translational modifications in RBP, and local enrichment of RNA close to membrane compartments [reviewed in (32)]. RNA sorting into EV may additionally be modulated by signaling processes triggered in the parental cells. There is strong evidence that miRNA sorting is influenced by cell stimulation (9, 16). Our own laboratory recently showed that EV-mediated release of Y-RNA is influenced by immune-related stimuli imposed on EV-producing cells (16). The EV-associated changes in Y-RNA were not reflected in cellular Y-RNA levels, which suggests that the Y-RNA shuttling rate, rather than the transcriptional level of Y-RNA, is modulated by these stimuli. Condition-dependent changes in the levels of extracellular Y-RNA have also been observed *in vivo*. Physical exercise was shown to increase the levels of circulating Y4-RNA, while Y1, Y3, and Y5 were decreased relative to resting conditions (103). Further research is needed to evaluate whether regulation of Y-RNA shuttling to the extracellular space is driven by differential expression or localization of Y-RNA binding proteins.

In conclusion, a large number of proteins known to interact with Y-RNA have been detected in EV. Some of these proteins may be involved in sorting of Y-RNA into EV, but the underlying mechanisms should be further explored (Figure 1, box 10). The co-presence of Y-RNA and Y-RNA binding proteins in EV also highlights the need to study the functional effects of EV-associated Y-RNA in the context of its protein partners.

## Y-RNA and Viruses

Extracellular Y-RNA has not only been detected in EV and RNP, but also in various retroviruses such as murine leukemia virus (MLV) and human immunodeficiency virus (HIV) (104–108) (Figure 1, box 11). These viruses incorporate not only Y-RNA, but also various other host-derived non-coding RNAs, such as tRNA and 7SL. The presence of extracellular Y-RNA in both retroviruses and EV is interesting because both structures are formed via overlapping biogenesis routes (109, 110). In addition, several Y-RNA binding proteins that have been identified in EV also interact with retroviral RNA. In the case of HIV, these proteins included YBX1, hnRNP K, PTBP1, Nucleolin, and Matrin-3 (111). This raises the interesting question whether there is overlap in mechanisms underlying the sorting of RNAs into EV and retroviruses.

Retroviruses use specific host tRNAs to prime reverse transcription, which is a key step in the retroviral life cycle (112). In addition, encapsidated host non-coding RNAs may mediate packaging of antiviral proteins, such as the antiviral cytidine deaminase APOBEC3G into virions (113). It has been hypothesized that newly synthesized host RNAs, including Y-RNAs that have not been bound by Ro60, may act as a scaffold for virion assembly (105, 106, 114). Moreover, it has been suggested that Y-RNA may benefit the host via potential triggering of TLR7 in newly infected cells, thereby initiating an antiviral immune response (107). Additionally, it was reported that many packaged RNAs, including Y-RNA, can mediate APOBEC packaging which leads to mutations in the viral genome or restricts retrotransposition (94). It remains to be investigated whether these Y-RNA-driven processes only occur during virus infection and whether we could learn from retroviruses to further delineate the function of Y-RNA in EV.

## The Role of Extracellular Y-RNA in Immune Regulation

The high abundance of Y-RNA in EVs and RNPs raises the question whether extracellular Y-RNAs have signaling functions when transferred to target cells (Figure 1, box 12). In general, it is technically challenging to assess the role of individual RNAs in EV-mediated effects because EV mediate simultaneous transfer of multiple proteins, lipids and RNAs to target cells. Until now, functional transfer of EV-associated miRNAs have been addressed either by using target cells with luciferase reporter constructs containing the 3′UTR of the mRNA target, or by investigating EV released by miRNA knockout cells, or by assessing the effects of transfecting synthetic analogs of the RNA of interest into target cells [reviewed in (32)]. It is unlikely that, upon transfer to target cells, Y-RNA functions similar to miRNA, as it has been shown that Y-RNA fragments bound to Ago2 were unable to repress reporter mRNAs (79). In addition, the effects of Y-RNA in EV may differ from those elicited by Y-RNA-containing RNP. Although the number of studies addressing the effects of extracellular Y-RNA are limited, the majority of these studies described effects of Y-RNA on immune regulation. Interestingly, both pro- and anti-inflammatory effects have been described, which will be discussed in more detail below.

Table 4 summarizes the immune-related effects that have been reported for extracellular Y-RNA subtypes in various experimental settings. Some of these studies specifically addressed the function of Y-RNA containing RNP (23, 115, 116), whereas others focused on the function of EV-associated RNA (24, 25). In the recent study by Haderk et al. it was shown that Y4 and 5′-fragments of Y4 were abundantly present in EV released by B cell leukemia cells. These EV not only induced inflammatory effects, such as the release of CCL2, CCL4 and IL6 by monocytes, but also induced PD-L1 expression on these cells, which inhibits T-cell activation (117). To investigate whether the EV-induced effects were mediated by Y4, monocytes were transfected with synthetic homologs of full-length Y4 or fragments thereof. Full length Y4, but not Y4 fragments, induced similar pro- and anti-inflammatory effects in monocytes as those observed after incubation with EV. Based on these data it was suggested that Y-RNA in tumor EV could contribute to establishing a favorable tumor microenvironment via suppressing the immune system (25).

**Table 4:** Overview of immune-related effects of extracellular Y-RNA.

**References**	**Source of Y-RNA**	**Y-RNA subtype**	**Approach**	**Recipient cell type**	**Immune-related effect**	**Conclusion**
Clancy et al. (115)	*In vitro* transcribed RNA and Ro60/Y-RNA complexes assembled *in vitro*	Y3-RNA	DOTAP transfection of *in vitro* transcribed RNA, and addition of Ro60/Y-RNA complexes to medium	Human macrophages; Fetal cardiac cells	macrophages: TNFa release, cardiac fibroblasts: collagen secretion	Increased TNFa release in macrophages; Increased collagen secretion by cardiac fibroblasts
Greidinger et al. (116)	*In vitro* transcribed RNA	All Y-RNAs	Addition to medium	RL-95 epithelial cells and HEK293 transiently transfected with TLR reporter constructs	RL-95: release of IL6, TLR-reporters: increased luciferase release,	Y-RNAs differ in their capacity to stimulate various RNA-sensing TLRs; Y1 stimulates TLR7 whereas Y3 stimulates TLR3
Hizir et al. (23)	Affinity purification from lysates from 293T cells treated with and without staurosporine (induces cleavage of Y-RNA)	Not specified	Addition to medium	Mouse and human monocytes/ macrophages	Apoptosis (caspase-3 cleavage, IkBa)	Cleaved Y-RNA associated with Ro60 induces inflammation and apoptosis, while naked Y-RNA does not. TLR7 triggering is involved
Cambier et al. (24)	Synthetic Y-RNA	Y4-RNA fragment	Transfection (Dharmafect 4 reagent)	Bone-marrow derived macrophages	mRNA expression (Arg1, IL4RA, Nos2, IL10, NFkB, TNF, TGFb, Vegfa) and increased IL10 release	Transfection of Y4-fragment in BMDM leads to prolonged induction of IL10
Haderk et al. (25)	EV isolated from MEC1 cell line by ultracentrifugation; Synthetic Y-RNA	Y4-RNA	Transfection (Effectene)	Monocytes	Cytokine release (CCL2, CCL4, IL-6), increased levels of surface markers (PD-L1, CCR2)	Transfected Y4-RNA or Y4-RNA enclosed in EV induces anti-inflammatory PD-L1

Y4-RNA containing EV have also been implicated in myocardial infarctions (24). Cardiosphere-derived cells (CDC) can reduce damage during myocardial infarction by modulating inflammatory responses via an unknown mechanism. It was found that CDC-EVs contain a relatively large percentage of Y-RNAs, and that one specific 5′ fragment of Y4-RNA was particularly abundant in CDC-EV compared to normal human dermal fibroblasts. Evidence was provided that EV could transfer Y4-fragments to bone-marrow derived macrophages, and that transfection of this Y4-fragment into macrophages resulted in strong and prolonged upregulation of IL10, and to a lesser extent TNFα. Additionally, administration of this Y4-fragment *in vivo* induced IL10 release and reduced damage in a myocardial infarction model in rats (24). Thus, the abundance of Y4-fragments in CDC-EVs correlated with the potency of these RNA fragments to mitigate damage after myocardial infarction.

The function of EV-associated Y-RNA has until now been addressed by transfecting Y-RNA (fragments) into target cells as a model for EV-mediated transfer of these RNAs. Although this may currently be the most feasible approach, several limitations can be identified. The naked, synthetic RNAs employed in these studies are not complexed to proteins, whereas RBP may play a role in the function of truly EV-associated Y-RNAs. Additionally, the route of uptake of lipofected RNA complexes may be different from EV-enclosed RNAs, resulting in delivery of the Y-RNA to subcellular locations other than those reached after EV-mediated delivery.

A few other studies provide indirect support for a role of EV-associated Y-RNA in immune-modulatory processes. For instance, Y-RNAs and tRNAs are particularly abundant in seminal plasma EV (prostasomes) (20), which are known to confer immune-suppressive effects leading to reduced rejection of sperm cells (118). Similarly, EV released by the parasite *Heligmosomoides polygyrus* contain high levels of nematode stem-bulge RNAs (which are related in sequence to Y RNAs) and suppress cytokine release in mice (119). Furthermore, in our latest study, we demonstrate that EV released from dendritic cells with an immune-suppressive function are more enriched in Y-RNA than EV released by dendritic cells with an immune-activating phenotype (16).

While EV-associated Y-RNAs seem to induce a range of different immune-related effects, circulating RNP containing Y-RNA predominantly induce immune activation. Many of these effects reportedly depend on TLR-mediated triggering (Figure 1, box 13). However, Y-RNA subtypes differ in their capacity to trigger different TLRs. Y3-RNA predominantly triggers TLR3, while Y1, Y3, and Y4 trigger TLR7 (116). Whereas, unbound Y-RNA may trigger TLR signaling, Y-RNA bound to protein partners such as Ro60 and La has a reduced stimulatory potential, likely because these proteins shield dsRNA hairpin structure and 5′ triphosphate group that are ligands for TLR and other pattern recognition receptors. In support of this idea, Clancy et al. showed that Ro60-associated Y3-RNA, in contrast to naked Y3-RNA, does not induce TNFα release in macrophages (115). The pro-inflammatory effects of Y-RNA-Ro60 complexes in autoimmune diseases such as SLE and SS are likely explained by binding of auto-antibodies to these RNP. Opsonization of Ro60-associated Y3-RNA by anti-Ro60 IgG was shown to be required for stimulation of TNFα release by macrophages, supporting a role for FcγR in this process (115) (Figure 1, box 14). However, not only FcγR-mediated triggering, but also RNA-mediated triggering of TLR7 contributed to the inflammatory effects elicited by the RNP (115). It is not known whether exposed Y-RNA-Ro60/La complexes only occur as RNP or whether these complexes are also present on the surface of EV. During apoptosis of fibroblasts, Y3-RNA was shown to drive the translocation of Ro60 to the outer leaflet of the plasma membrane (120) (Figure 1, box 15). Upon opsonization with anti-Ro60 antibodies, these apoptotic fibroblasts induced TNFα release in macrophages in a TLR7 dependent manner (120). Since apoptotic cells release various types of EV as well as apoptotic bodies (121), it is possible that some of these EV display surface-exposed Y-RNA-Ro60 complexes. In fact, it is known that EV associate with autoantibodies in several autoimmune diseases, thereby forming proinflammatory complexes that contribute to disease [reviewed in (122)].

Besides triggering inflammation in SLE and SS, extracellular Y-RNA complexes have also been demonstrated to induce apoptosis in atherosclerosis (23) and cancer (80). In atherosclerosis, lipoproteins accumulating in arteries can lead to activation of macrophages and subsequent apoptosis induction in these cells. *In vitro* cultured macrophages treated with lipids release increased levels of fragmented Y-RNA into the medium (22). Hizir et al. showed that affinity purified Y-RNA fragments/Ro60 RNPs from apoptotic HEK293T cells induced cell death in macrophages (Figure 1, box 16). Y-RNA fragment-containing RNP released by macrophages could therefore contribute to a negative feed-back loop in which more and more macrophages in the lipid-rich environment die by apoptosis. In the context of cancer, it was shown that EV released by myelogenous leukemia cell lines contain high levels of fragmented Y5-RNA (80). Not only these EV, but also deproteinized total RNA from these EV and synthetic Y5 fragments were shown to induce apoptosis in healthy cells, but not in cancer cells. Via this mechanism, Y5-fragments in EV could favor cancer cell proliferation and invasion of tissues.

The studies described above suggest that the functional effects of extracellular Y-RNAs depend on both the macromolecular structure to which it is associated and the conditions under which the Y-RNA is released. In addition to the TLR-mediated effects of Y-RNA that have been reported to date (Figure 1, box 17), Y-RNA may also mediate functional effects via their interacting proteins (Figure 1, box 18). This highlights the importance of separating EV from RNP in studies addressing the function of EV-associated RNA (32, 123).

## Biomarker Potential of Extracellular Y-RNA

The abundance of circulating Y-RNA in body fluids has triggered interest in the potential use of Y-RNA as biomarker for disease. Increased levels of Y-RNA have been observed in the circulation of cancer patients (14, 21, 25, 31). In breast cancer patients, the abundance of 3′ Y-RNA fragments was higher than in healthy controls (21). A more recent study on chronic lymphocytic leukemia (CLL) reported the increased abundance of Y4-RNA in serum from CLL patients compared to healthy controls (25). However, these studies were performed with small groups of patients and the data currently lack power to confirm the suitability of Y-RNA (fragments) as biomarkers for cancer. Whereas there is no evidence that EV from tumor cells are more enriched in Y-RNA than their non-tumorigenic counterparts, most tumor cells release relatively high numbers of EV (14, 25). The cancer-related increase in circulating Y-RNA may therefore be explained by increased numbers of tumor cell-derived EV in the circulation. Alternatively, other cell types may react to the presence of the tumor by increasing cellular export of Y-RNA.

Increased levels of circulating extracellular Y-RNA have also been observed in the context of atherosclerosis and coronary artery disease. Repetto and colleagues observed a higher number of 5′ Y1-fragments in the blood of ApoE^−/−^mice used as a model for atherosclerosis (22). Likewise, increased levels of circulating 5′-Y1 were observed in a cohort of 43 men with stable coronary artery disease (CAD), as compared to 106 age-matched healthy men. These data were validated in an independent sub-cohort including 220 patients vs. 408 controls. In 45 CAD patients an increased abundance of Y4-RNA 5′-fragments was observed (22). This raises the question of which cells are the main producers of extracellular Y-RNA fragments present in the circulation. The suggested candidates include macrophages (23) and platelets (81). However, it is important to note that pre-analytical variables can strongly affect characterization of extracellular RNA in plasma. Plasma samples are commonly contaminated by platelets, which may disintegrate during freezing (34), thereby releasing their internal (RNA) content. Indeed, plasma miRNA levels were shown to correlate with platelet counts prior to freezing (124). Thus, there is an urgent need for standardization of sample collection, storage conditions and sample processing for reliable assessment of Y-RNA and other extracellular RNAs present in body fluids.

In conclusion, differences in circulating Y-RNA may be further explored as biomarkers for disease, but it is critically important to evaluate and standardize the various methods used to isolate different carriers of Y-RNA in body fluids. Additionally, acquisition of knowledge on how disease-associated changes in cells affect the release of Y-RNAs will help to better understand their biomarker potential.

## Concluding Remarks

Current data suggest that the family of Y-RNAs does not only play a role in intracellular processes to maintain cell function, but also acts as versatile intercellular messengers. Various studies have indicated that extracellular transport of Y-RNA is a highly efficient process employed by many different cell types. Additionally, Y-RNA is one of the most abundant extracellular non-coding RNAs in human plasma. Such extracellular RNA can occur in RNP or in EV. One potential trigger that regulates extracellular release of Y-RNA is TLR signaling. Moreover, currently available data suggest functional involvement of extracellular Y-RNA in various immune-related processes. Y-RNAs can bind to several different proteins. We here provided an overview of Y-RNA binding proteins that occur inside cells and in Y-RNA-containing RNP or EV released by cells into the extracellular milieu. We propose that binding to these proteins not only determines how Y-RNA regulates cellular processes, but may also drive their sorting into EV and could be essential for functional effects of Y-RNA transferred to recipient cells (Figure 1). Partly based on currently available data, we envision that Y-RNA may affect the function of recipient cells via different mechanisms. These include direct effects of Y-RNA, such as activation of RNA sensors (e.g., TLRs), leading to the release of pro- and anti-inflammatory cytokines. Additional effects may be mediated by Y-RNA binding proteins, many of which function in regulation of transcription and translation. Initial data suggest that levels of extracellular Y-RNA may correlate with disease. However, more research is needed as to how Y-RNA release is altered in diseased cells and how this affects other cells in order to delineate the contribution of extracellular Y-RNA in (immune-related) diseases and to correctly interpret its applicability as a disease biomarker.

## Author Contributions

EN performed literature research, drafted the manuscript, and edited the text. TD performed literature research, made the inventory of Y-RNA binding proteins in EV by searching data repositories, and wrote the manuscript.

### Conflict of Interest Statement

The authors declare that the research was conducted in the absence of any commercial or financial relationships that could be construed as a potential conflict of interest.
